# The effects of online pilates and face-to-face pilates in healthy individuals during the COVID-19 pandemic: a randomized controlled study

**DOI:** 10.1186/s13102-023-00625-3

**Published:** 2023-02-02

**Authors:** Halil I. Bulguroglu, Merve Bulguroglu

**Affiliations:** Faculty of Health Sciences, Department of Physiotherapy and Rehabilitation, Ankara Medipol University, Ankara, Turkey

**Keywords:** COVID-19, Core muscle endurance, Depression, Online exercise, Pilates

## Abstract

**Background:**

Along with the covid-19 process, people started to turn to online exercise methods. One of these methods is the pilates method, which increases the endurance of the core muscles. This study aims to analyze and compare the effects of online and face-to-face pilates methods.

**Methods:**

Fifty-eight healthy individuals aged 25–40 years were included in the study. Individuals were randomly divided into three groups; online pilates group (OPG), face-to-face pilates group (FPG), and control group (CG). Pilates groups were given pilates exercises in groups of three or four for eight weeks, three days a week, for 1 h a day, by the physiotherapist. The control group did breathing and relaxation exercises at home. Core muscular endurance, depression, and quality of life were assessed before and after eight weeks of training.

**Results:**

Core muscle endurance, depression, and quality of life improved after pilates in online and face-to-face pilates groups (*p* < 0.05). No change was found in the control group (*p* > 0.05). When the gains in the Pilates groups were compared, it was seen that the improvement in the Modified Biering-Sorensen test was more significant in the face-to-face pilates group, and the improvement in the trunk flexion test was more significant in the online group (*p* < 0.05), while the gains in other parameters were similar (*p* > 0.05).

**Conclusions:**

As a result, healthy individuals have seen similar benefits in online and face-to-face pilates. Both methods are significant for gaining healthy habits and increasing physical activity in healthy individuals.

*Trial registration* Retrospectively registered. NCT05309486, Registration date: 04/04/2022. URL: https://www.clinicaltrials.gov/ct2/show/NCT05309486?term=BULGUROGLU&draw=2&rank=1

## Background

Telerehabilitation; is a developing method that reduces service barriers such as time, cost, and distance, eliminates the financial burden, provides savings, and brings individuals and healthcare professionals together in a virtual environment.

It has been shown in the literature that the tele-exercise services provided as part of telerehabilitation services, which became widespread during the Covid -19 pandemic process, have beneficial effects in increasing the decreasing physical activity and reducing the emerging problems [1, 2].

The pandemic process we have been through has provided us health professionals with the opportunity to spread online methods. One of the most important and easy ways to gain healthy habits and keep all the body's systems, but perhaps the most important one, the immune system, is to exercise. In addition, considering these effects in the post-pandemic period, it is thought that online methods can continue to be used [3, 4]. Another exercise method used as tele-exercise in the literature is Pilates [5]. Developed by Joseph Pilates in the 1900s, pilates is an increasingly common exercise method that increases the strength, endurance, and flexibility of the trunk stabilizer muscles. The method focuses heavily on mind–body integrity [6, 7].

Studies on Pilates in the literature show that face-to-face Pilates training positively affects core muscle endurance, mental health, and quality of life in healthy individuals [8, 9].

Several recent studies have examined the effects of online pilates training and have been shown to affect core endurance and quality of life [5, 10]. In the results of these studies, it was emphasized that online pilates training is applicable; On the other hand, it was stated that online training should be compared with whether it is as practical as face-to-face pilates training.

Therefore, we thought there was a need for randomized controlled studies investigating the effects of online and face-to-face pilates in healthy individuals. We planned a randomized controlled study that examined the effects of online pilates and face-to-face pilates methods. Our study aims to analyze and compare the effects of online pilates and face-to-face pilates methods on core muscle endurance, depression, and quality of life in healthy individuals.

## Methods

### Participants

We conducted the study at Ankara Medipol University, Faculty of Health Sciences, Department of Physical Therapy and Rehabilitation. Before starting the study, ethical approval was obtained from Ankara Medipol University Non-Interventional Clinical Research Ethics Committee with the decision dated 24.12.2021 and numbered 65.

All methods were performed following the relevant CONSORT 2010 guidelines and regulations (Clinical Trials.gov Number NCT05309486, First Registration 04/04/2022) and were conducted according to the Declaration of Helsinki.

Sixty-four healthy university workers aged 25–40 were voluntarily included in the study. The exclusion criteria for this study were: any cardiovascular, orthopedic, visual, hearing, and perception problems that may affect the research results and participation in other exercises or physiotherapy programs during the past six months.

### Procedures

Evaluations at the beginning of the study and after eight weeks of training; were performed by an experienced physiotherapist blinded to randomization. The group they belonged to was not disclosed to the participants until the end of the baseline assessment. We used a simple method (Microsoft Excel 2016) for randomization. After the initial evaluation, individuals were randomly assigned to online pilates group (OPG), face-to-face pilates group (FPG), or control group (CG), unaware of the person performing the statistical analysis. We completed Pilates exercises under the supervision of a certified experience physiotherapist in both pilates groups. We asked individuals not to inform the evaluator in which group they were in the final evaluation. Before the study, we explained the purpose and content of the study to the participants and obtained written informed consent for participation from all participants.

### Intervention

Online pilates and face-to-face pilates training, which lasted three days a week, 1 h for eight weeks, was carried out by Australian Pilates and Physiotherapy Institute certified and experienced Ph.D. Physiotherapist Halil Ibrahim Bulguroglu. While the Microsoft Teams program was used to apply the online pilates method, face-to-face pilates training was held at Ankara Medipol University. In both groups, exercises were performed three days a week, at the same time as possible, in the afternoon. Our study in both groups started on March 15, 2022, and was completed on May 24, 2022. To perform the exercises more effectively and efficiently, we divided the individuals in both pilates groups into small groups. Pilates practitioners typically perform a simple series of low-intensity, repetitive exercises involving the trunk, hip, thigh, and arm movements to increase endurance [6]. The same exercises were applied in both pilates groups, including hundreds, single leg circles, shoulder bridge, single leg stretch, clam, sidekick, side leg lifts, arms opening, breaststroke preparations, swan, swimming, and the roll-up. In both groups, exercises were performed as 15 min of warm-up, 30 min of pilates, and 15 min of cooling and stretching exercises. For both groups, the basic elements of the first session of pilates; breathing, focus, rib cage position, shoulder position, and head and neck positions were taught, and Individuals were asked to maintain the smoothness of these key elements throughout the exercises. Different imagery techniques were used in the exercises, and the exercises were combined with breathing exercises. In addition, visual and verbal imagery techniques were used during the exercises in both groups. Individuals in both groups were carefully observed during the exercise, and necessary corrections were made in order for them to perform the movements correctly. Subjects in both pilates groups were informed about side effects such as shortness of breath, dizziness, headache, muscle pain, and weakness. They were asked to stop exercising when they experienced any of these side effects. Subjects in the control group were asked to follow a home program of relaxation and breathing exercises three times a week for eight weeks. After the initial assessment, a program of approximately 50 min consisting of diaphragmatic breathing, pursed lip breathing, and respiratory control was given to the individuals as a home program.

### Outcome measurements

Participants included in the study were evaluated with data collection forms filled twice, before and after the training programs. We recorded the participants' demographic information (age, body weight, height, body mass index).

We evaluated core muscle endurance with the Side Bridge test, Modified Biering-Sorensen test, Trunk Flexion test, and Prone Bridge test [11]. Measurements that ended after 30 s or when subjects changed their test positions were recorded with a stopwatch as seconds. In the side bridge test, the subjects were asked to form a straight line by lifting their body off the ground on their right forearm and feet. It was repeated on the other side.

In the modified Biering-Sorensen test, while an assistant supported the individual's pelvis on a workout bench, the individual was asked to raise the upper body hanging from the workout bench to the level of the bench. In the trunk flexion test, subjects were held with their backs angled 60° from the ground. In the prone bridge test, subjects were asked to keep their bodies straight, with their feet hip-width apart, toes, and elbows. In all tests, the time elapsed until the individuals broke the test position was recorded with a stopwatch.

We evaluated depression with Beck Depression Inventory (BDI) [12]. The score range in BDI, a 21-item self-evaluation type scale, is 0–63. For the BDI, a score of 17 to 29 indicates mild depression, and 30 or above indicates severe depression. The Turkish version of the Beck Depression Inventory was used in our study [13]. The Cronbach’s alpha value of Hisli’s validity and reliability study was 0.74, and that of this study was 0.827.

We evaluated Quality of Life with Short Form-36 (SF-36) [14]. SF-36; consists of 36 items to measure eight sub-parameters: physical, mental, and general health. Cronbach's alpha value of Ware’s validity and reliability study was between 0.62 and 0.94, and that of this study was 0.71 and 0.89. In the subscales evaluated between 0 and 100, a high score indicates a good quality of life. Our study used the Turkish version of the Short Form-36 [15].

### Statistical analysis

Statistical analysis was performed using SPSS software, version 26 (SPSS Inc. Chicago, IL, USA). The normal distribution of variables was determined using histograms, probability plots, and the Shapiro–Wilk test. Because of an abnormal distribution, median and interquartile range (IQR) were used for descriptive statistics. Mann–Whitney U test is used for demographic characteristics. One-way repeated measures ANOVA was used in the analysis of variables measured repeatedly (pre and post-test) between groups. Also, Bonferroni post-hoc test was used to determine the source of the difference between the groups. The significance level was determined as *p* < 0.05 and *p* < 0.01.

## Results

**Fig. 1:**
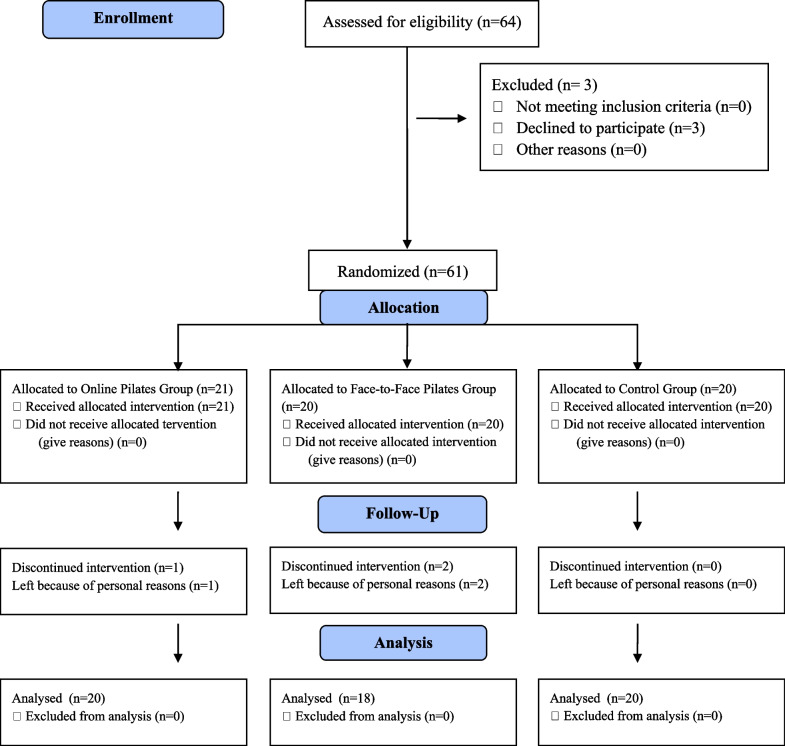
CONSORT flowchart showing participation in the study

Within the scope of the study, we invited 64 healthy participants for evaluation. Three participants were excluded: those who withdrew at their request. The remaining sixty-one participants were allocated to three subgroups using a simple randomization method. The first group included 21 participants who received online pilates exercises; the second group had 20 participants who received face-to-face pilates exercises, and the third group, as a control group, including 20 participants. One participant in the first group and two in the second group left the study for personal reasons. Consequently, 20 subjects from OPG, 18 from FPG, and 20 from CG completed the study. Figure 1 shows the CONSORT flow diagram showing the participants in the study.

In the post hoc power analysis for the study, the effect size calculated considering the depression values was 1.076. The power of the study was calculated as 0.84 when 20 subjects were included in OPG and 18 subjects in FPG.

The patients’ demographic characteristics can be seen in Table 1.

**Table 1:** Demographic characteristics of the groups

	Online Pilates Group Median (IQR) n = 20	Face-to-Face Pilates Group Median (IQR) n = 18	Control Group Median (IQR) n = 20	p
Age (years)	28 (25–31)	29 (26–33)	27 (24–32)	0.749
Height (cm)	171 (155–179)	170 (154–178)	168 (156–176)	0.317
Weight (kg)	71 (53–95)	72 (55–94)	73 (55–94)	0.658
BMI-1 (kg/ cm^2^)	22.34 (19.93–32.78)	23.14 (18.79–29.85)	22.76 (17.61–26.75)	0.128
BMI-2 (kg/ cm^2^)	22.94 (18.33–33.25)	22.58 (19.24–30.13)	22.33 (18.17–29.73)	0.471

Data are presented as Median (IQR). cm: centimeters, kg: kilograms, BMI: body mass index

When looking at Table 2, there was a statistically significant difference with a moderate effect size between pre-and post-test measurement times for Side bridge R (F(_3,60_) = 23,613, *p* < 0.001, η2 = 0.062) and L (F(_3,60_) = 26,125, *p* < 0.001, η2 = 0.059) tests of participants. In addition, it was determined that there was a statistically different with a large effect size between the Side bridge R (F(_2,42_) = 11,638; *p* < 0.01, η2 = 0.171) and Side bridge L (F(_2,42_) = 3149; *p* < 0.01, η2 = 0.159) of the pilates groups’. According to this result, it was determined that there was a difference between the control group and the Side bridge R and Side bridge L values of the OPG and FPG groups (*p* < 0.01), while there was no difference between the OPG and FPG groups (*p* > 0.05). In addition, the highest increase in Side bridge R (226.38%) and Side bridge L tests (175.29%) was in FPG.

**Table 2:** A comparison of the previous and subsequent measurement of core endurance and depression parameters for groups

Variable	Group	Pre-test (mean ± SD)	Post-test (mean ± SD)	∆ (%)	F	*P*
CORE ENDURANCE						
Side bridge (s) R	OPG	14.13 ± 3.29	28.16 ± 2.97	99.29	11638	< 0.001
	FPG	8.11 ± 4.37	26.47 ± 4.13	226.38		
	CG	11.26 ± 3.86	12.31 ± 2.29	9.33		
		F = 23,613; *p* < 0.001			Interaction F = 4161; *p* = 0.325	
Side bridge (s) L	OPG	13.16 ± 2.89	26.54 ± 2.71	101.67	3149	< 0.001
	FPG	9.27 ± 4.26	25.52 ± 4.25	175.29		
	CG	8.81 ± 3.68	7.16 ± 2.91	− 18.72		
		F = 26,125;* p* < 0.001			Interaction F = 14,151; *p* = 0.216	
Modified Biering-Sorensen (s)	OPG	28.42 ± 4.98	43.76 ± 4.71	53.98	6641	< 0.001
	FPG	29.25 ± 5.82	59.43 ± 4.25	103.18		
	CG	27.54 ± 5.17	26.16 ± 3.26	− 5.01		
		F = 81,245; * p* < 0.001			Interaction F = 24,821; * p* < 0.001	
Trunk flexion (s)	OPG	7.81 ± 3.81	25.17 ± 3.17	222.28	17,372	< 0.001
	FPG	5.25 ± 4.57	19.62 ± 4.28	273.71		
	CG	6.16 ± 3.24	5.24 ± 5.13	− 14.94		
		F = 27,237; * p* < 0.001			Interaction F = 14,262; * p* < 0.001	
Prone bridge (s)	OPG	18.27 ± 3.64	38.52 ± 4.29	110.84	15,147	< 0.001
	FPG	21.45 ± 3.38	32.79 ± 2.76	52.87		
	CG	18.71 ± 4.76	19.25 ± 3.81	2.89		
		F = 38,154; * p* < 0.001			Interaction F = 8162; *p* = 0.061	
DEPRESSION						
Beck Depression Inventory (0–63)	OPG	13.86 ± 4.74	9.24 ± 4.69	− 33.33	6741	< 0.001
	FPG	12.47 ± 6.64	8.62 ± 5.31	− 30.87		
	CG	12.15 ± 5.35	13.51 ± 3.26	11.19		
		F = 11,894; * p* < 0.001			Interaction F = 8741; *p* = 0,278	

SD: Standart deviation. OPG: Online pilates group; FPG: Face-to-face pilates group, CG: Control group

In Table 2 examined, there was a statistically significant difference with a moderate effect size between pre-and post-test measurement times for the Modified Biering-Sorensen (F(_3,60_) = 81,245; *p* < 0.01, η2 = 0.083) test of participants. In addition, it was determined that there was a statistically difference with a large effect size between Modified Biering-Sorensen of the pilates groups’ (F(_2,42_) = 6641; *p* < 0.01, η2 = 0.22). According to this result, it was determined that there was a difference between the control group and the Modified Biering-Sorensen values of the OPG and FPG groups (*p* < 0.01), and there was a difference in favor of FPG between the OPG and FPG groups (*p* < 0.05). In addition, the highest increase in Modified Biering-Sorensen test (103.18%) was in FPG.

When Table 2 examined, there was a statistically significant difference with a moderate effect size between pre-and post-test measurement times for trunk flexion (F(_3,60_) = 27,237; *p* < 0.01, η2 = 0.078) test of participants. In addition, it was determined that there was a statistically difference with a large effect size between the pilates groups’ trunk flexion (F(_2,42_) = 17,372; *p* < 0.01, η2 = 0.413). According to this result, it was determined that there was a difference between the control group and the trunk flexion values of the OPG and FPG groups (*p* < 0,01), and there was a difference in favor of OPG between the OPG and FPG groups (*p* < 0,05). In addition, the highest increase in trunk flexion test (273.71%) was in FPG.

In Table 2 examined, there was a statistically significant difference with a moderate effect size between pre-and post-test measurement times for the Prone bridge (F(_3,60_) = 38,154; *p* < 0.01, η2 = 0.096) test of participants. In addition, it was determined that there was a statistically difference with a large effect size between the Prone bridge of pilates groups’ (F(_2,42_) = 15,147; *p* < 0.01, η2 = 0.482). According to this result, it was determined that there was a difference between the control group and the Prone bridge values of the OPG and FPG groups (*p* < 0.01), while there was no difference between the OPG and FPG groups (*p* > 0.05). In addition, the highest increase in the Prone bridge test (110.84%) was in OPG.

When looking at Table 2, there was a statistically significant difference with a moderate effect size between pre-and post-test measurement times for the Beck Depression Inventory (F(_3,60_) = 11,894; *p* < 0.01, η2 = 0.071) test scores of participants. In addition, it was determined that there was a statistically difference with a large effect size between Beck Depression Inventory test scores of pilates groups’ (F(_2,42_) = 6741; *p* < 0.01, η2 = 0.235). According to this result, it was determined that there was a difference between the control group and the Beck Depression Inventory test scores of the OPG and FPG groups (*p* < 0.01). At the same time, there was no difference between the OPG and FPG groups (*p* > 0.05). In addition, the highest reduction in Beck Depression Inventory test scores (33.33%) was in OPG.

When Table 3 was examined, there was a statistically significant difference with a moderate effect size between pre-and post-test measurement times for Physical functioning subscale scores (F(_3,60_)=16,107; *p *< 0.01, η2 = 0.063) of participants. In addition, it was determined that there was a statistically difference with a large effect size between Physical functioning subscale scores of pilates groups’ (F(_2,42_)=12,265; *p*< 0.01, η2 = 0.208). According to this result, it was determined that there was a difference between the control group and the Physical functioning subscale scores of the OPG and FPG groups (*p *< 0.01), while there was no difference between the OPG and FPG groups (*p *> 0.05). In addition, the highest increase in Physical functioning subscale scores (6.20%) was in OPG.

**Table 3:** A comparison of the previous and subsequent measurement of quality of life parameters (physical functioning, role limitations, vitality) for groups

Variable	Group	Pre-test (mean ± SD)	Post-test (mean ± SD)	∆ (%)	F	P
*Quality of life (short form-36)*						
Physical functioning	OPG	85.13 ± 4.26	90.41 ± 4.22	6.20	12,265	< 0.001
	FPG	85.64 ± 3.28	90.72 ± 4.81	5.93		
	CG	85.12 ± 4.21	85.61 ± 3.74	0.58		
		F = 16,107; * p* < 0.001			Interaction F = 5178; *p* = 0.421	
Role limitations (physical problems)	OPG	85.74 ± 4.65	94.27 ± 2.28	9.95	14,275	< 0.001
	FPG	85.76 ± 2.88	93.76 ± 4.16	9.33		
	CG	80.71 ± 3.74	70.52 ± 7.13	− 12.63		
		F = 19,351; * p* < 0.001			Interaction F = 6139; *p* = 0.275	
Role limitations (emotional problems)	OPG	80.24 ± 3.75	98.74 ± 1.25	23.06	15.548	< 0.001
	FPG	90.71 ± 3.38	97.76 ± 2.58	7.78		
	CG	80.15 ± 2.79	79.71 ± 3.74	− 0.55		
		F = 26,575; * p* < 0.001			Interaction F = 7168; *p* = 0.061	
Vitality	OPG	60.12 ± 2.55	70.34 ± 2.35	17.01	14,981	< 0.001
	FPG	58.94 ± 2.71	69.26 ± 1.97	17.51		
	CG	67.81 ± 5.27	58.71 ± 2.14	− 13.42		
		F = 18,127; * p* < 0.001			Interaction F = 3237; *p* = 0.452	

SD: Standart deviation. OPG: Online pilates group; FPG: Face-to-face pilates group, CG: Control group

In Table 3 examined, there was a statistically significant difference with a moderate effect size between pre-and post-test measurement times for Role limitations (physical problems) subscale scores (F(_3,60_) =19,351; *p *< 0.01, η2 = 0.091) of participants. In addition, it was determined that there was a statistically different with a large effect size between Role limitations (physical problems) subscale scores of pilates groups’ (F(_2,42_)=14,275; *p *< 0.01, η2 = 0.018). According to this result, it was determined that there was a difference between the control group and the Role limitations (physical problems) subscale scores of the OPG and FPG groups (*p *< 0.01). At the same time, there was no difference between the OPG and FPG groups (*p *> 0.05). In addition, the highest increase in Role limitations (physical problems) subscale scores (9.95%) was in OPG.

When looking at Table 3, there was a statistically significant difference with a moderate effect size between pre-and post-test measurement times for Role limitations (emotional problems) subscale scores (F(_3,60_)=26,575; *p *< 0.01, η2 = 0.082) of participants. In addition, it was determined that there was a statistically different with a large effect size between Role limitations (emotional problems) subscale scores of pilates groups’ (F(_2,42_)=15,548; *p *< 0.01, η2 = 0.213). According to this result, it was determined that there was a difference between the control group and the Role limitations (emotional problems) subscale scores of the OPG and FPG groups (*p *< 0.01). At the same time, there was no difference between the OPG and FPG groups (*p *> 0.05). In addition, the highest increase in Role limitations (emotional problems) subscale scores (23.06%) was in OPG.

When Table 3 was examined, there was a statistically significant difference with a moderate effect size between pre-and post-test measurement times for Vitality subscale scores (F(_3,60_)=18,127; *p *< 0.01, η2 = 0.012) of participants. In addition, it was determined that there was a statistically different with a large effect size between Vitality subscale scores of pilates groups’ (F(_2,42_) =14,981; *p *< 0.01, η2 = 0.264). According to this result, it was determined that there was a difference between the control group and the Vitality subscale scores of the OPG and FPG groups (*p *< 0.01), while there was no difference between the OPG and FPG groups (*p* > 0.05). In addition, the highest increase in Vitality subscale scores (17.51%) was in FPG.

When looking at Table 4, there was a statistically significant difference with a moderate effect size between pre-and post-test measurement times for Mental health (F(_3,60_) = 28,813; *p* < 0.01, η2 = 0.062) and Social functioning (F(_3,60_) = 34,504; *p* < 0.01, η2 = 0.088) subscale scores of participants. In addition, it was determined that there was a statistically different with a large effect size between Mental health (F(_2,42_) = 11,651; *p* < 0.01, η2 = 0.171) and Social functioning (F(_2,42_) = 21,248; *p* < 0.01, η2 = 0.185) subscale scores of pilates groups’. According to this result, it was determined that there was a difference between the control group and the Mental health and Social functioning subscale scores of the OPG and FPG groups (*p* < 0.01), while there was no difference between the OPG and FPG groups (*p* > 0.05). In addition, the highest increase in Mental health (11.99%) and Social functioning subscale scores (17.24%) was in FPG.

**Table 4:** A comparison of the previous and subsequent measurement of quality of life parameters (mental health, social functioning, pain, general health perception) for groups

Variable	Group	Pre-test (mean ± SD)	Post-test (mean ± SD)	∆ (%)	F	P
*Quality of life (short form-36)*						
Mental health	OPG	75.74 ± 6.11	84.14 ± 3.43	11.09	11,651	< 0.001
	FPG	74.76 ± 5.34	83.73 ± 2.91	11.99		
	CG	76.71 ± 6.18	79.12 ± 2.84	3.14		
		F = 28,813; * p* < 0.001			Interaction F = 1237; *p* = 0.327	
Social functioning	OPG	74.12 ± 3.97	86.12 ± 2.87	16.19	21.248	< 0.001
	FPG	73.28 ± 2.37	85.91 ± 4.12	17.24		
	CG	78.13 ± 4.12	74.51 ± 2.13	− 4.63		
		F = 34,504; * p* < 0.001			Interaction F = 2136; *p* = 0.094	
Pain	OPG	75.45 ± 3.75	88.47 ± 4.85	17.26	20,236	< 0.001
	FPG	76.89 ± 3.48	87.75 ± 5.48	14.13		
	CG	58.13 ± 5.74	58.51 ± 3.91	0.65		
		F = 27,087; * p* < 0.001			Interaction F = 4478; *p* = 0.151	
General health perception	OPG	68.34 ± 6.21	79.58 ± 4.11	16.45	9151	< 0.001
	FPG	63.96 ± 3.45	77.34 ± 3.48	20.92		
	CG	69.91 ± 5.84	68.21 ± 4.13	− 2.43		
		F = 29,476; *p* < 0.001			Interaction F = 0.476; *p* = 0,237	

SD: Standart deviation. OPG: Online pilates group; FPG: Face-to-face pilates group, CG: Control group

When Table 4 was examined, there was a statistically significant difference with a moderate effect size between pre-and post-test measurement times for Pain subscale scores (F(_3,60_) = 27,087; *p* < 0.01, η2 = 0.108) of participants. In addition, it was determined that there was a statistically different with a large effect size between Pain subscale scores of pilates groups’ (F(_2,42_) = 20,236; *p* < 0.01, η2 = 0.242). According to this result, it was determined that there was a difference between the control group and the Pain subscale scores of the OPG and FPG groups (*p* < 0.01), while there was no difference between the OPG and FPG groups (*p* > 0.05). In addition, the highest increase in Pain subscale scores (17.26%) was in OPG.

In Table 4 examined, there was a statistically significant difference with a moderate effect size between pre-and post-test measurement times for General health perception subscale scores (F(_3,60_) = 29,476; *p* < 0.01, η2 = 0.092) of participants. In addition, it was determined that there was a statistically different with a large effect size between General health perception subscale scores of pilates group’. (F(_2,42_) = 9151; *p* < 0.01, η2 = 0.514). According to this result, it was determined that there was a difference between the control group and the General health perception subscale scores of the OPG and FPG groups (*p* < 0.01), while there was no difference between the OPG and FPG groups (*p* > 0.05). In addition, the highest increase in General health perception subscale scores (20.92%) was in FPG.

## Discussion

This study answered, "Is there a difference between core muscle endurance, depression, and quality of life development due to online and face-to-face pilates training in healthy individuals?". We found that online pilates and face-to-face pilates training improved all these parameters, and online pilates exercises were at least as practical as face-to-face pilates training.

Our study found that the core muscle endurance of healthy individuals increased in both online and face-to-face pilates groups. Although one study in the literature shows the effect of online pilates training on core muscle endurance in healthy individuals [5], many studies show that face-to-face pilates exercise improves core muscle endurance in healthy individuals [17, 18].

Keklik et al., In a study completed with 33 healthy participants, stated that 6-week online pilates training improved all core muscle endurance tests.

A systematic review included nine randomized controlled studies discussing the effect of face-to-face pilates exercises on healthy individuals. It was concluded that pilates exercises performed two or three times a week for 5–12 weeks increased core muscle endurance [17]. Another systematic review included 16 randomized controlled trials and concluded that Pilates training provides moderate evidence for improving muscular endurance in healthy individuals [18].

The endurance of the core muscles is significant in the successful performance of daily life activities and the continuation of healthy life [19]. Pilates, which provides core muscle training in three planes, focuses on trunk stabilization [20–22].

We think the increase in core endurance with pilates is related to the increase in the transversus abdominis muscle activity, which is the primary muscle of the "core" region and which we activate with the centering principle of pilates.

When we look at the differences in the gains obtained with pilates training in both pilates groups, it was observed that there was a statistically significant increase in favor of face-to-face pilates training in the Modified Biering-Sorensen test and in favor of online pilates training in the trunk flexion test. Although there was no statistically significant difference in any other parameter between face-to-face and online application, the highest change from baseline occurred in the face-to-face group. This more improvement in the face-to-face pilates training group, the closer the instructor is to the participant, although the basic principle is the same, making the person feel safe. In light of the results of our study, it was concluded that both pilates training could be used to develop core muscle endurance. Moreover, face-to-face pilates training may be more effective than online pilates training.

Our study observed that depression levels decreased in the eight weeks of pilates training.

Although our study is the first to show the effect of online pilates training on depression levels in healthy individuals, many studies show that pilates exercise reduces depression levels in healthy individuals [23–25].

A systematic review that included eight randomized controlled trials said the available evidence is that pilates training effectively promotes mental health [23]. Hassan and Amin stated in their study that the increase in serotonin levels with pilates exercises could help in reducing the symptoms associated with depressive symptoms and pessimistic attitudes [24]. Another study stated that pilates focused on breathing exercises, positively affecting emotional states such as depression in the elderly [25].

The results of all these studies are similar to our results. In our study, with both pilates training, thanks to principles such as concentration, breathing, and integrated isolation, we saw that participants' self-perceptions improved and their self-confidence increased. We think that these effects of both pilates training reduce the depression levels of participants. While the current pandemic caused people to stay away from physical activity and social participation, it also caused depression levels to increase. We think that being in contact with other participants and not feeling lonely with pilates training is also effective in reducing depression levels. In addition, regular exercise during the pandemic process may have made the participant feel safer and more robust and positively affected depression levels. 

When we compared the gains in depression levels in online pilates training and face-to-face pilates training groups, it was seen that the gains in both groups were almost similar. This result shows us that both methods can improve depression levels in healthy individuals.

Our study improved the participants’ quality of life in both pilates groups.

No studies in the literature show the effects of online pilates training on the quality of life in healthy individuals. However, it has been stated in previous studies that pilates training improves the quality of life in healthy individuals [26–29]. Studies have shown that pilates training improves the quality of life in healthy individuals and individuals with any pathology.

Similar to other studies showing improvement in our study, an increase in the quality of life of individuals in both pilates groups was observed. We think that this increase makes significant contributions to the lives of individuals. Although we think these developments are closely related to the development in the level of depression, we think that the physiological benefits of exercise and the individual’s self-confidence by exercising may also be effective in this result.

When the groups’ improvements in the quality of life after pilates training were compared, no significant difference was found. We think that both pilates training methods can be used to improve the physical and mental quality of life of individuals.

During the study, we had the chance to observe the advantages and disadvantages of both pilates methods. The face-to-face pilates method is a method that allows people to observe more closely and correct movement with tactile feedback when necessary. Also, the person and the trainer are closer to exercise development. Thus, further development can be achieved in the face-to-face pilates method, thanks to the trainer's assistance. However, the face-to-face pilates method is a more expensive practice and has disadvantages such as place and time.

On the other hand, the online pilates method is a method that eliminates problems such as place and time and even eliminates the need to be in the same city. Thus, it becomes easier for trainers to reach individuals. It allows individuals to do exercises in their environment. However, it is necessary to be careful, especially in exercises where tactile stimulation is required and in the development of the exercises.

One of the strengths of our study is that it is a randomized controlled evaluator blinded study. In addition, another strength of our work is that the exercises are done regularly and in small groups in both groups, in a specific discipline, at the same time every week, in company with a physiotherapist. The fact that both groups received pilates training by the same person may be a limitation of our study.

## Conclusions

The pandemic process allows us to increase individuals’ physical activity and give them healthy habits. In this study, it has been shown that online pilates training is as practical as face-to-face pilates training, is easy to apply, and can be used after the pandemic process.

As a result, similar benefits were seen in healthy people in both pilates methods. While deciding which of these training may be more suitable for individuals, the individual's preferences and possibilities should be considered. We think that our study offers physiotherapists an online pilates training method that can be used during and after the pandemic, at least as effective as face-to-face pilates, easy to apply, and an exercise method that individuals can continue throughout their lives.

## Data Availability

The datasets generated during and analyzed during the current study are not publicly available due to confidential information about the participants but are available from the corresponding author on reasonable request at [fztibrahim@hotmail.com].

## Abbreviations

BDI: Beck depression ınventory
BMI: Body mass index
CG: Control group
CM: Centimeters
FPG: Face-to-face pilates group
IQR: Interquartile range
KG: Kilograms
L: Left
OPG: Online pilates group
R: Right
SF-36: Short form-36
